# Disparities in cancer survival and incidence by metropolitan versus rural residence in Utah

**DOI:** 10.1002/cam4.1382

**Published:** 2018-03-13

**Authors:** Mia Hashibe, Anne C. Kirchhoff, Deanna Kepka, Jaewhan Kim, Morgan Millar, Carol Sweeney, Kimberley Herget, Marcus Monroe, N. Lynn Henry, Ana‐Maria Lopez, Kathi Mooney

**Affiliations:** ^1^ Department of Family and Preventive Medicine Huntsman Cancer Institute University of Utah School of Medicine Salt Lake City Utah; ^2^ Department of Pediatrics Huntsman Cancer Institute University of Utah School of Medicine Salt Lake City Utah; ^3^ College of Nursing Huntsman Cancer Institute University of Utah Salt Lake City Utah; ^4^ College of Health University of Utah Salt Lake City Utah; ^5^ Division of Epidemiology Department of Internal Medicine University of Utah School of Medicine Salt Lake City Utah; ^6^ Utah Cancer Registry University of Utah Salt Lake City Utah; ^7^ Division of Otolaryngology Department of Surgery Huntsman Cancer Institute University of Utah School of Medicine Salt Lake City Utah; ^8^ Division of Oncology Department of Internal Medicine Huntsman Cancer Institute University of Utah School of Medicine Salt Lake City Utah

**Keywords:** 5‐Year survival, cancer incidence, disparity, endometrial cancer, rural

## Abstract

Cancer disparities in rural and frontier communities are an important issue in Utah because much of Utah is sparsely populated. The aims of this study were to investigate whether there are differences in the cancer incidence and 5‐year survival rates in Utah by metropolitan/rural residence and to investigate disparities in distributions of cancer risk factors. We used cancer registry records to identify patients diagnosed with a first primary cancer in Utah between 2004 and 2008. We estimated 5‐year survival and incidence rates. The Cox proportional hazards model was used to estimate hazard ratios (HRs) for the risk of death. There were 32,498 (86.9%) patients with cancer who lived in metropolitan counties and 4906 (13.1%) patients with cancer who lived in rural counties at the time of cancer diagnosis. Patients with cancer from rural counties were more likely to be older, American Indian/Alaskan Native, non‐Hispanic, male, and diagnosed at higher stage. Rural residents had a five‐year relative survival that was 5.2% lower than metropolitan residents and a 10% increase in risk of death (HR = 1.10, 95% CI = 1.03, 1.18) after adjustment for multiple factors. Overall, the cancer incidence rates in rural counties were lower by 11.9 per 100,000 per year (449.2 in rural counties vs. 461.1 in metropolitan counties). Cancer patients living in rural counties of Utah had different demographic characteristics as well as differences in incidence and survival rates. Further studies with individual‐level data are necessary to investigate the reasons behind these differences in cancer incidence and survival to reduce disparities.

## Introduction

Rural areas in the United States have higher incidence and death rates for tobacco‐ and screening‐related cancers based on Surveillance, Epidemiology, and End Results (SEER) data 1. Although the cancer incidence rate declines were similar in metropolitan and rural areas, the cancer mortality rate declines were less in rural areas. In the Appalachia region, rural residents had the highest cancer incidence rates as well as cancer mortality rates 2. Approximately 16,000 rural Appalachian cancer patients would not have died if the rural regions had experienced mortality rates similar to the urban areas over a 4‐year period. A study of 3562 endometrial cancer patients from the Kentucky cancer registry reported that rural endometrial cancer survivors had higher proportions of uninsured, black race, and unknown stage compared to urban survivors 3. They were less likely to have received combination therapy of surgery and radiation, and more likely to receive treatment in small hospitals. Disease‐specific survival however did not differ between rural and urban endometrial cancer patients.

Utah is an ideal location to assess cancer disparities among individuals living in rural and frontier communities because the vast majority of Utah is sparsely populated or considered “frontier.” Approximately 70% of Utah's 80,000 square miles have less than seven persons per square mile which is considered frontier 4. Although previous studies have reported on differences in incidence and death rates by metropolitan and rural regions, the Utah population is unique with lower tobacco use, lower alcohol drinking prevalence, higher educational attainment, lower obesity rates, and a younger population compared to other states in the United States 5. With decreasing tobacco prevalence in the United States, we might expect that the Utah population represents where the general US population will be in another 10 years regarding tobacco‐related cancers. For colorectal cancer in Utah, the incidence was higher among nonmetropolitan women between 2006 and 2010 and nonmetropolitan women also did not experience increased survival 6. Additionally, to our knowledge, previous studies have not incorporated tobacco, obesity, and other factors to investigate survival differences between rural vs. urban cancer patients.

Although previous studies found potential disparities in cancer survival and incidence between rural and urban residents, it is critical to further examine whether there are differences in the cancer incidence and 5‐year survival rates in Utah by metropolitan/rural residence and to investigate possible factors such as stage at diagnosis, cigarette smoking, obesity, and screening behaviors for any differences in survival observed using SEER data. County‐level variables from SEER have not been utilized extensively for these purposes.

## Methods

We used the SEER 18 database 7 and included cancer patients with the following eligibility criteria: (1) diagnosed between 2004 and 2008 to allow for estimations of minimum 5‐year survival time since follow‐up was available up to 2013; (2) diagnosis in Utah as identified by the Utah cancer registry; (3) first primary cancer diagnosis; and (4) age at diagnosis 20+ years old. We used the county characteristics discussed below to group cancer patients in metropolitan and rural residence at cancer diagnosis. There were a total of 37,404 eligible cancer patients. We also assessed age at diagnosis, race, Hispanic ethnicity, sex, and stage at diagnosis (derived SEER summary stage 2000 for 2004+ diagnoses). Insurance status was available for patients with cancer diagnosed in 2007–2008 for part of the cancer patient group (*n* = 15,711).

The 29 counties in Utah were categorized as metropolitan or rural. The “Metropolitan Statistical Area” designation is determined by the Office of Management and Budget as “based on urbanized areas of 50,000 or more population.” “A Metropolitan Statistical Area containing a single urbanized area with a population of at least 2.5 million may be subdivided to form smaller groupings of counties referred to as Metropolitan Divisions. A county qualifies as a “main county” of a Metropolitan Division if 65% or more of workers living in the county also work within the county and the ratio of the number of workers working in the county to the number of workers living in the county is at least .75. 8” From the SEER data, metropolitan counties included (1) counties in metro areas of 1 million population or more, (2) counties in metro areas of 250,000 to 1 million population, and (3) counties in metro areas of fewer than 250,000 population. Rural counties included (1) urban population of 20,000 or more, adjacent to a metro area, (2) urban population of 20,000 or more, not adjacent to a metro area, (3) urban population of 2500 to 19,999, adjacent to a metro area, (4) urban population of 2500 to 19,999, not adjacent to a metro area, (5) completely rural or less than 2500 urban population, adjacent to a metro area, and (6) completely rural or less than 2500 urban population, not adjacent to a metro area. There were 32,498 patients with cancer who lived in metropolitan counties and 4906 patients with cancer who lived in rural counties at the time of cancer diagnosis.

For county‐level variables, we selected the data available that were closest to the years of diagnosis for the cancer patient cohort (2004–2008). County‐level variables from SEER*Stat, originating from the 2000 US Census, included education (% at least Bachelor degree 2000), income (median family income 2000), and poverty (% families below poverty 2000). County‐level variables from SEER*Stat that was based on the Behavioral Risk Factor Surveillance System (BRFSS) and the National Health Interview Survey (NHIS) included smoking status (% current Smoker, age 18+, 2000–2003), pap smear (% pap smear within 3 years, age 18+ 2000–2003), and mammography (% mammography within 2 years, age 40+ 2000–2003). The proportion of obese individuals by county was obtained from publicly available data from the Utah State Health Department [https://opendata.utah.gov/Health/Adult-Obesity-Rates-By-County-In-Utah-2010-2016/ynme-hu3g/data].

### Statistical analysis

We used SAS Cary, North Carolina, US version 9.1 to analyze the data for survival. We used the Chi‐square test to assess differences in the distribution of demographic, clinical, and county‐level variables between metropolitan residents and rural residents. The Kaplan–Meier (KM) curve was used to compare the probability of survival between the two groups, and the log‐rank test was used to compare overall survival functions in the KM curve. The Cox proportional hazards model was used to estimate hazard ratios (HRs) for the risk of death. Time to event was calculated as the time from cancer diagnosis to cancer death or end of follow‐up. Five‐year relative survival rates were estimated using SEER*Stat version 8.3.4. We selected cancer sites with at least 100 metropolitan cancer cases and 50 rural cancer cases to assure sufficient sample sizes to calculate relative survival rates.

We adjusted for age, sex, and race/ethnicity when assessing the potential association between rural residence at cancer diagnosis and the risk of death as those factors are risk factors for death and may impact decisions on living in a rural county (i.e., they meet the three properties of confounders 9). Other prognostic factors such as stage at cancer diagnosis, cancer treatment, and county‐level education, income, obesity, and smoking were adjusted on as sensitivity analyses to assess the impact on the hazard ratio.

For incidence analysis, we included patients diagnosed between 2009 and 2013 to investigate a recent cohort. The eligibility criteria were as follows: (1) diagnosis in Utah, (2) first primary cancer diagnosis, and (3) age at diagnosis 20+ years old. We used SEER*Stat version 8.3.4 for incidence rates that were age‐adjusted, using the 2000 US standard population from the census with 19 age groups.

## Results

Demographic and clinical characteristics for the patients with cancer from metropolitan vs. rural counties are shown in Table 1. Patients with cancer from rural counties were more likely to be older, American Indian/Alaskan Native, non‐Hispanic, male, and diagnosed at higher stage. For cancer treatment, rural residents were more likely to have no radiation/surgery and have missing data for treatment. The proportion of cancer patients with insurance was lower among the rural residents (73.8% in rural vs. 77.4% in metropolitan).

**Table 1:** Characteristics of Utah cancer patients diagnosed 2004–2008 in metropolitan and rural counties

	Metropolitan counties (*n* = 32,498)		Rural counties (*n* = 4,906)		*P*‐value for Chi‐square
	*n*	%	*n*	%	
Age at diagnosis					
<50	6546	20.1	737	15.0	<0.0001
50–64 years	10,776	33.2	1631	33.2	
65+ years	15,176	46.7	2538	51.7	
Race					
White	31,566	97.1	4,785	97.5	<0.0001
Black	198	0.6	6	0.1	
American Indian/Alaska Native	92	0.3	85	1.7	
Asian or Pacific Islander	593	1.8	18	0.4	
Unknown	49	0.2	12	0.2	
Hispanic					
No	30,759	94.6	4764	97.1	<0.0001
Yes	1739	5.4	142	2.9	
Sex					
Male	17,315	53.3	2739	55.8	0.0008
Female	15,183	46.7	2167	44.2	
Stage					
In situ	3234	10.0	416	8.5	0.0003
Localized	16,085	49.5	2,335	47.6	
Regional	6240	19.2	933	19.0	
Distant	5869	18.1	974	19.9	
Unknown	1070	3.3	248	5.1	
Treatment					
No radiation or surgery	7018	21.6	1,186	24.2	<0.0001
Radiation only	3439	10.6	600	12.2	
Surgery only	16,695	51.4	2434	49.6	
Radiation and surgery	4889	15.0	571	11.6	
Unknown	457	1.4	115	2.3	
Insurance status (for 2007–8; *n* = 15,711)					
Uninsured	331	2.4	55	2.8	<0.0001
Any medicaid	493	3.6	106	5.3	
Insured	8850	64.5	1086	54.8	
Insured/no specifics	1270	9.3	271	13.7	
Insurance status unknown	2785	20.3	464	23.4	

Surveillance, Epidemiology, and End Results (SEER) Program (www.seer.cancer.gov) SEER*Stat Database: Incidence – SEER 18 Regs Research Data + Hurricane Katrina Impacted Louisiana Cases, Nov 2015 Sub(1973–2013 varying) – Linked To County Attributes – Total U.S., 1969–2014 Counties, National Cancer Institute, DCCPS, Surveillance Research Program, Surveillance Systems Branch, released April 2016, based on the November 2015 submission.

For the cancer patients living in rural counties, the proportion completing a bachelor or college degree in the year 2000 was lower, median income was lower, proportions of families living below poverty were higher, percent obese was higher, and percent current smoking was higher (Table 2). For cancer screening, both pap smears (within 3 years) and mammography (within 2 years) were less likely in rural counties.

**Table 2:** County‐level characteristics of Utah cancer patients diagnosed 2004–2008 in metropolitan and rural counties

	Metropolitan counties (*n* = 32,498)		Rural counties (*n* = 4906)		*P*‐value for Chi‐square
	*n*	%	*n*	%	
% Bachelors degree 2000					
<15	106	0.3	1644	33.5	<0.0001
15–24	6977	21.5	2978	60.7	
>24	25,414	78.2	284	5.8	
Median family income 2000 ($)					
<50,000	4052	12.5	3845	78.4	<0.0001
≥50,000	28,446	87.5	1061	21.6	
% Families below poverty 2000					
<7	4592	14.1	309	6.3	<0.0001
7–9	23,854	73.4	1147	23.4	
>9	4052	12.5	3450	70.3	
% Obese 2010					
<23	716	2.2	1357	27.7	<0.0001
23–25	27,577	84.9	1418	28.9	
>25	4205	12.9	2131	43.4	
% Current smoker 2000‐2003					
<15	10,657	32.8	1063	21.7	<0.0001
15–17	3488	10.7	1040	21.2	
>17	18,353	56.5	2803	57.1	
% Pap smear within 3 years 2000–2003 (age 18+; *n* = 17,350 women)					
<75	1127	7.4	1649	76.1	<0.0001
≥75	14,056	92.6	518	23.9	
%Mammography within 2 years 2000–2003 (age 40+; *n* = 17,350 women)					
<65	7430	48.9	1746	80.6	<0.0001
≥65	7753	51.1	421	19.4	

Surveillance, Epidemiology, and End Results (SEER) Program (http://www.seer.cancer.gov) SEER*Stat Database: Incidence – SEER 18 Regs Research Data + Hurricane Katrina Impacted Louisiana Cases, Nov 2015 Sub (1973–2013 varying) – Linked To County Attributes – Total U.S., 1969–2014 Counties, National Cancer Institute, DCCPS, Surveillance Research Program, Surveillance Systems Branch, released April 2016, based on the November 2015 submission.

For all cancer sites combined, rural residents had a five‐year relative survival that was 5.2% lower than metropolitan residents (Table 3). The cancers for which rural residents experienced lower survival were brain cancer (6.6% lower), endometrial cancer (6.6% lower), oral cavity and pharyngeal cancer (5.7% lower), and kidney and renal pelvis cancer (5.1% lower). Slightly higher five‐year relative survival rates were experienced by rural residents for ovarian cancer (2.0% higher), thyroid cancer (1.2% higher), and pancreatic cancer (0.8% higher). Survival curves for all cancers combined and the most common cancers are shown in Figure S1 for metropolitan and rural cancer patients.

**Table 3:** Five‐year relative survival rates for Utah cancer patients diagnosed 2004–2008 in metropolitan and rural counties

	Metropolitan counties (*n* = 29,451)		Rural counties (*n* = 4295)		Difference in survival rates (metropolitan – rural)^a^
	*N*	5‐Year survival (95% CI)	*N*	5‐Year survival (95% CI)	
All sites	29,451	75.0 (74.4–75.6)	4295	69.8 (68.1–71.4)	5.2
Oral cavity and pharynx	540	69.0 (64.2–73.2)	87	63.3 (50.8–73.5)	5.7
Colon and rectum	2530	67.8 (65.6–70.0)	406	64.9 (59.0–70.1)	2.9
Pancreas	667	7.3 (5.4–9.7)	98	8.1 (3.5–15.1)	−0.8
Lung and bronchus	1766	16.0 (14.2–17.9)	363	14.0 (10.5–18.1)	2.0
Melanoma of the Skin	2084	92.7 (90.8–94.3)	290	91.5 (85.1–95.2)	1.2
Female breast	4303	88.9 (87.6–90.2)	538	87.1 (82.8–90.4)	1.8
Cervix uteri	237	71.3 (64.5–76.9)	50	71.1 (55.8–81.9)	0.2
Corpus and uterus, NOS	920	87.8 (84.7–90.3)	113	81.2 (70.7–88.2)	6.6
Ovary	450	44.0 (39.1–48.8)	55	46.0 (31.9–59.0)	−2.0
Prostate	6484	99.9 (99.1–100)	944	97.9 (95.2–99.1)	2.0
Urinary bladder	995	77.5 (73.5–81.0)	163	74.4 (63.7–82.3)	3.1
Kidney and renal pelvis	786	73.3 (69.4–76.8)	116	68.2 (57.5–76.7)	5.1
Brain	477	32.2 (28.0–36.5)	64	25.6 (15.4–37.0)	6.6
Thyroid	1243	98.6 (97.3–99.3)	125	99.8 (0.0–100.0)	−1.2
Lymphoma	1651	76.2 (73.6–78.5)	207	74.7 (67.2–80.8)	1.5
Myeloma	369	44.1 (38.3–49.7)	70	44.0 (31.1–56.1)	0.1
Leukemia	857	56.4 (52.5–60.2)	155	54.1 (44.7–62.6)	2.3

a A positive number indicates a higher survival rate in metropolitan counties.

Rural residence was associated with an 7% increase in risk of death (Table 4). The increased risk of death was persistent after adjustment for most factors and all factors together (10% increase). Other prognostic factors associated with increased risks of death among the entire cohort of cancer patients included older age at diagnosis, black or unknown race/ethnicity, being male, and higher stage at diagnosis. Compared to patients with cancer who did not receive radiation or surgery, patients who had received any combination of radiation and surgery were protected against the risk of death. Increased risk of death was observed for patients who lived in counties with a higher percentage of current smokers (HR = 1.06, 95% CI = 1.02–1.11; Table 5). We observed a decreased risk of death for women in counties with higher mammography rates.

**Table 4:** Risk of death among cancer among Utah residents diagnosed 2004–2008

	Subjects	Death	Hazard ratio^f^	95% CI
Rural^a^				
Metropolitan	32,408	11,911	1.00	
Rural	4887	2074	**1.07**	**(1.03**–**1.13)**
Rural residence, with additional adjustments^a^				
Stage			**1.07**	**(1.02**–**1.12)**
Education			**1.06**	**(1.00**–**1.13)**
Income			**1.11**	**(1.05**–**1.18)**
Obesity			**1.07**	**(1.02**–**1.12)**
Smoking			**1.07**	**(1.02**–**1.12)**
Radiation and surgery^b^			**1.06**	**(1.01**–**1.12)**
Full model			**1.10**	**(1.03**–**1.18)**
Other factors				
Age at diagnosis^c^				
<50	7274	1188	1.00	
50–64 years	12,377	3349	**1.79**	**(1.66**–**1.92)**
65+ years	17,644	9448	**4.08**	**(3.82**–**4.36)**
Race^d^				
White	36,248	13,520	1.00	
Black	201	88	**1.54**	**(1.23**–**1.91)**
Other	786	335	**1.29**	**(1.14**–**1.45)**
Unknown	60	42	**1.82**	**(1.34**–**2.48)**
Hispanic^d^				
No	35,421	13,303	1.00	
Yes	1874	682	**1.21**	**(1.11**–**1.31)**
Sex^d^				
Male	19,995	7808	1.00	
Female	17,300	6177	**0.94**	**(0.91**–**0.98)**
Stage^e^				
In situ	3650	561	1.00	
Localized	18,408	4202	**1.89**	**(1.71**–**2.08)**
Regional	7168	2922	**3.70**	**(3.34**–**4.11)**
Distant	6824	5256	**10.75**	**(9.66**–**11.96)**
Treatment^e^				
No radiation or surgery	8204	5566	1.00	
Radiation only	4039	1815	**0.71**	**(0.67**–**0.76)**
Surgery only	19,129	4951	**0.28**	**(0.27**–**0.30)**
Radiation and surgery	5460	1350	**0.32**	**(0.30**–**0.35)**

a Adjusted on age, sex, race/ethnicity, and cancer site.

b No treatment, radiation only, surgery only, radiation, and surgery.

c Adjusted on age, sex, race/ethnicity, rural residence, county‐level education, county‐level income, and cancer site.

d Adjusted on age.

e Adjusted on age, sex, race/ethnicity, rural residence, county‐level income, and cancer site.

f Statistically significant HRs and 95%CIs are in bold

**Table 5:** County‐level characteristics and the risk of death among Utah cancer patients diagnosed 2004–2008

	Subjects	Death	Hazard ratio^a^	95% CI
% Bachelors degree 2000				
<15	1742	790	1.00	
15–24	9924	4038	0.97	(0.89–1.07)
>24	25,629	9157	0.94	(0.85–1.05)
Median family income 2000				
<50,000	7869	3210	1.00	
≥50,000	29,246	10,775	1.04	(0.97–1.13)
% Families below poverty 2000				
<7	4893	1642	1.00	
7–9	24,927	9302	**1.07**	**(1.01–1.13)**
>9	7475	3041	1.17	(0.99–1.40)
% Obese 2010				
<23	2067	763	1.00	
23–25	28,913	10,668	0.99	(0.90–1.07)
>25	6315	2554	0.94	(0.84–1.05)
% Current smoker 2000–2003				
<15	11,693	4023	1.00	
15–17	4513	1870	1.03	(0.96–1.12)
>17	21,089	8092	**1.06**	**(1.02–1.11)**
% Pap smear within 3 years 2000–2003 (*n* = 17,350 women)				
<75	2766	1163	1.00	
≥75	14,534	5014	0.93	(0.86–1.01)
% Mammography within 2 years 2000–2003 (age 40+; *n* = 17,350 women)				
<65	9,149	3303	1.00	
≥65	8151	2874	**0.95**	**(0.92–0.99)**

a Adjusted for metro/rural residence, age, sex, race/ethnicity, education, income, and cancer site where appropriate.

Overall, the cancer incidence rates in rural counties were lower by 11.9 per 100,000 per year (449.2 in rural counties vs. 461.1 in metropolitan counties; Table 6). Higher cancer incidence rates were observed for metropolitan counties for cancers of the pancreas (2.1 per 100,000 higher), thyroid (5.1 per 100,000 higher), female breast (6.3 per 100,000 higher), and prostate (21.1 per 100,000 higher). Higher cancer incidence rates were observed in rural counties for lung cancer (5.6 per 100,000 higher) and colorectal cancer (4.5 per 100,000 higher).

**Table 6:** Age‐adjusted incidence rates for Utah cancer patients diagnosed 2009–2013 in metropolitan and rural counties

	Metropolitan counties (*n* = 33,323)		Rural counties (*n* = 4470)		Difference (rural – metropolitan)^a^
	Rate (95% CI)	Count	Rate (95% CI)	Count	
All sites	461.1 (456.1–466.2)	33,323	449.2 (436.5–462.2)	4740	−11.9
Oral cavity and pharynx	8.9 (8.2–9.6)	648	8.3 (6.7–10.3)	94	−0.6
Colon and rectum	35.4 (34–36.9)	2,518	39.9 (36.2–43.9)	411	4.5
Pancreas	12.5 (11.7–13.4)	870	10.4 (8.6–12.6)	110	−2.1
Lung and bronchus	29.7 (28.4–31)	2040	35.3 (31.8–39)	373	5.6
Melanoma of the Skin	36.7 (35.3–38.1)	2694	34 (30.5–37.8)	385	−2.7
Breast	131.6 (127.9–135.4)	4905	125.3 (115.8–135.3)	645	−6.3
Cervix uteri	6.4 (5.7–7.3)	249	8.5 (6.1–11.5)	41	2.1
Corpus and uterus, NOS	31.4 (29.6–33.2)	1,216	32.9 (28.2–38.1)	171	1.5
Ovary	13.2 (12–14.4)	496	12.2 (9.4–15.6)	67	−1
Prostate	186.9 (182.2–191.7)	6330	165.8 (155.1–177)	882	−21.1
Urinary bladder	16.9 (15.9–17.9)	1144	15.4 (13.1–18)	156	−1.5
Kidney and renal pelvis	14.2 (13.3–15.1)	1035	15.5 (13.2–18)	161	1.3
Brain	7 (6.4–7.6)	535	8.8 (7.1–10.9)	86	1.8
Thyroid	23.7 (22.6–24.8)	1865	18.6 (16–21.4)	197	−5.1
Lymphoma	23.1 (22–24.2)	1684	21.4 (18.6–24.4)	219	−1.7
Myeloma	7 (6.4–7.7)	470	6 (4.6–7.6)	75	−1
Leukemia	12.5 (11.7–13.4)	901	14.3 (12.1–16.8)	144	1.8

Rates are per 100,000 and age‐adjusted to the 2000 US Std Population (19 age groups – Census P25‐1130) standard. Surveillance, Epidemiology, and End Results (SEER) Program (http://www.seer.cancer.gov) SEER*Stat Database: Incidence – SEER 18 Regs Research Data + Hurricane Katrina Impacted Louisiana Cases, Nov 2015 Sub (1973–2013 varying) – Linked To County Attributes – Total U.S., 1969–2014 Counties, National Cancer Institute, DCCPS, Surveillance Research Program, Surveillance Systems Branch, released April 2016, based on the November 2015 submission.

a A negative number indicates a higher incidence rate in metropolitan counties.

## Discussion

Cancer patients living in rural counties of Utah had different distributions of demographic characteristics, lower cancer incidence rates, and lower survival rates. The greatest differences in survival between metropolitan and rural residents were observed for brain cancer, endometrial cancer, oral cavity and pharyngeal cancers, and kidney and pelvis cancer, with higher survival rates for all of these cancers among metropolitan residents. The greatest differences in incidence were observed for breast and prostate cancer, with higher rates among metropolitan residents. Overall, rural residence was associated with a 10% increase in the risk of death among cancer patients in Utah, which persisted after adjustment for various factors such as stage of cancer, treatment, obesity, education, income, and smoking.

Rural residents were diagnosed with cancer at an older age and later stage, perhaps due to differences in access to care by distance to primary care, major cancer hospitals as well as differences in insurance status. The screening rates for pap smears and mammography were also very different, with the majority of rural residents living in counties with low pap smear rates (<75%) and low mammography rates (<65%) among women. The corresponding incidence rates of screening‐related cancers including breast and prostate cancers were much higher in metropolitan regions. The survival rates for breast and prostate cancer were higher in metropolitan regions. These results suggest that the lower screening rates in rural counties may contribute to lower cancer incidence for some cancers, but the later diagnosis may result in lower survival.

Differences in treatment were observed between metropolitan and rural residents, with a higher proportion in the latter cohort receiving no surgery or radiation which may be appropriate if they were diagnosed at later stage and palliation is indicated. Adjustment for treatment did not impact the increase in the risk of death observed among rural residents, although we were only able to take into account radiation and surgery and not systemic therapies. Treatment differences alone may not explain the increase in the risk of death among metropolitan residents. However, treatment differences by rural and metropolitan counties are a concern, and further areas of study could include differences in adherence to treatment guidelines and barriers to obtaining treatment for patients.

Smoking prevalence was higher in the rural counties of Utah where patients with cancer lived. Cancer incidence rates of tobacco‐related cancers were generally higher in the rural counties. However, the overall cancer incidence rates were lower in rural counties in Utah, in contrast to results from the Appalachian rural region where cancer incidence rates were higher 2. The difference in incidence rates in rural Utah vs. rural Appalachia compared to their urban counterparts may be due to the lower tobacco prevalence in Utah, although tobacco use was higher in the Utah rural counties than Utah metropolitan counties. The lower screening mentioned earlier also may contribute to the lower overall cancer incidence rates in the rural areas of Utah compared to the metropolitan areas.

Obesity‐related cancers were not all higher in incidence rates among rural residents. For example, endometrial cancer (corpus and uterus) is one of the cancers most strongly associated with obesity, but there was no difference in the incidence rates between metropolitan and rural residents. In contrast, the largest difference in survival rates between rural and metropolitan regions was observed for endometrial cancer (6.6% higher in metropolitan regions). Survival rates for two other obesity‐related cancers, kidney cancer, and colorectal cancer were also higher in metropolitan regions. Overall risk of death for cancer patients was not clearly associated with obesity rates in the counties; this may be because we estimated the risk of death for cancer overall and the association of obesity with cancer survival appears to differ by cancer site 10. Higher obesity in rural counties needs to be addressed as a public health issue for cancer survivors especially given the data regarding better outcomes with weight loss.

The 10% increase in the risk of death among rural cancer patients persisted after adjustment for various factors. However, as some of the adjustment variables were at a county level and not individual level, residual confounding may be a limitation for this estimate. The estimate is also fairly modest, although it is still supportive of an increase in risk. Further studies focusing on individual cancers, with individual‐level data on education, obesity, smoking, and income, would be beneficial to clarify the contributions of those factors to the suggested increase in the risk of death among cancer patients living in rural regions.

The strengths of our study include the large sample size with investigation of various cancers. The SEER data provide the advantage of investigating metropolitan vs. rural differences with a population‐based design that covers the entire state of Utah. We explored obesity, income, and education as possible predictors for the differences in survival between rural vs. urban residents. We were able to investigate the unique Utah population which includes approximately 13% rural residents and has very low rates of tobacco use and obesity.

Our study also has some limitations such as ecologic bias due to the use of county‐level characteristics for obesity, income, and education. However, the county‐level data allowed us to explore the potential role of these factors in the risk of death among patients with cancer. The associations were largely in the direction expected showing that the county‐level variables are useful proxy measures. Additionally, although the overall sample size was large, we were unable to explore rarer cancers, and for some of the cancer investigated, the sample sizes in the rural populations were limited. We also assumed that rural residence at cancer diagnosis reflects general residential history without changes, but some patients with cancer may relocate due to treatment or for other reasons from rural to urban areas. More detailed studies including residential history would be beneficial to understand the contribution of rural residence to survival differences.

In summary, our study showed important differences in survival and cancer incidence between rural and metropolitan residents in Utah that were different from other rural populations in the United States. Further studies are necessary to investigate the reasons behind these differences with individual‐level data in cancer incidence and survival. The reasons for differences in treatment for rural vs. metropolitan cancer patients need to be studied in terms of treatment guideline adherence, access to care, and insurance status. The role of obesity and education in differences in survival by residence should be studied for individual cancers. Understanding the role of individual‐level factors in cancer survival differences will be crucial in eliminating these disparities for the future.

## Conflict of Interest

None declared.

## Supporting information


**Figure S1**. Survival curves for Utah Cancer patients diagnosed 2004–2008 (red line = Rural, blue line = Metropolitan; *P*‐value for log‐rank: all cancers *P* < 0.0001, prostate *P* = 0.4663, colon cancer *P* = 0.2596, breast cancer *P* = 0.0079, melanoma *P* = 0.2389, lung cancer *P* = 0.6542).

## References

[1] Henley, S. J. , R. N. Anderson , C. C. Thomas , G. M. Massetti , B. Peaker , and L. C. Richardson . 2017 Invasive Cancer Incidence, 2004–2013, and Deaths, 2006–2015, in Nonmetropolitan and Metropolitan Counties ‐ United States. MMWR Surveill. Summ. 66:1–13. 10.15585/mmwr.ss6614a1 PMC5879727 28683054

[2] Yao, N. , H. E. Alcalá , R. Anderson , and R. Balkrishnan . 2016 Cancer disparities in rural appalachia: incidence, early detection, and survivorship. J. Rural Health. 33:375–381. 27602545 10.1111/jrh.12213

[3] Modesitt, S. C. , B. Huang , B. J. Shelton , and S. Wyatt . 2006 Endometrial cancer in Kentucky: the impact of age, smoking status, and rural residence. Gynecol. Oncol. 103:300–306. 16631234 10.1016/j.ygyno.2006.03.009

[4] Utah Department of Health (UDOH) . Utah Rural Health Plan. 2013.

[5] Centers for Disease Control and Prevention , National Center for Chronic Disease Prevention and Health Promotion , Division of Population Health . BRFSS Prevalence & Trends Data [online]. 2015 Available at https://www.cdc.gov/brfss/brfssprevalence/ (accessed 17 February 2017).

[6] Fowler, B. , N. J. Samadder , D. Kepka , Q. Ding , L. Pappas , and A. C. Kirchhoff . 2017 Improvements in colorectal cancer incidence not experienced by nonmetropolitan women: a population‐based study from Utah. J. Rural Health. 2017 Apr 20. [Epub ahead of print]. 10.1111/jrh.12242 PMC5650577 28426915

[7] Surveillance, Epidemiology, and End Results (SEER) Program (http://www.seer.cancer.gov) SEER*Stat Database: Incidence – SEER 9 Regs Research Data, Nov 2014 Sub (1973–2012) <Katrina/Rita Population Adjustment> – Linked To County Attributes – Total U.S., 1969‐2013 Counties, National Cancer Institute, DCCPS, Surveillance Research Program, Surveillance Systems Branch, released April 2015, based on the November 2014 submission. 2015.

[8] Office of Management and Budget 2010 Standards for delineating metropolitan and micropolitan statistical areas. Fed. Reg. 2010:75.

[9] Rothman, K. J. 2008 Modern epidemiology, 3rd ed. Lippincott Williams & Wilkins, Philadelphia, PA.

[10] Demark‐Wahnefried, W. , E. A. Platz , J. A. Ligibel , C. K. Blair , K. S. Courneya , J. A. Meyerhardt , et al. 2012 The role of obesity in cancer survival and recurrence. Cancer Epidemiol. Biomarkers Prev. 21:1244–1259. 22695735 10.1158/1055-9965.EPI-12-0485 PMC3415558

