# Supplementary material for: Disparities in cancer survival and incidence by metropolitan versus rural residence in Utah
Source: Cancer Med. 2018 Mar 13;7(4):1490–7. doi: 10.1002/cam4.1382 (PMC5911626; doi:10.1002/cam4.1382)
Supplement: Supplementary file 1 — Figure S1. Survival curves for Utah Cancer patients diagnosed 2004–2008 (red line = Rural, blue line = Metropolitan; P‐value for log‐rank: all cancers P < 0.0001, prostate P = 0.4663, colon cancer P = 0.2596, breast cancer P = 0.0079, melanoma P = 0.2389, lung cancer P = 0.6542). [file CAM4-7-1490-s001.docx]

|  |  |
| --- | --- |
|  |  |
|  |  |

Supplemental Figure 1. Survival curves for Utah Cancer patients diagnosed 2004-2008 (red line=Rural, blue line=Metropolitan; p-value for log-rank: all cancers p<0.0001, prostate p=0.4663, colon cancer p=0.2596, breast cancer p=0.0079, melanoma p=0.2389, lung cancer p=0.6542)
